# Assessing the Relative Importance of Local and Regional Processes on the Survival of a Threatened Salmon Population

**DOI:** 10.1371/journal.pone.0099814

**Published:** 2014-06-12

**Authors:** Jessica A. Miller, David J. Teel, William T. Peterson, Antonio M. Baptista

**Affiliations:** 1 Department of Fisheries and Wildlife, Coastal Oregon Marine Experiment Station, Oregon State University, Newport, Oregon, United States of America; 2 Northwest Fisheries Science Center, NOAA Fisheries, Manchester, Washington, United States of America; 3 Northwest Fisheries Science Center, NOAA Fisheries, Newport, Oregon, United States of America; 4 NSF Science and Technology Center for Coastal Margin Observation and Prediction, Oregon Health and Science University, Beaverton, Oregon, United States of America; University of California, Berkeley, United States of America

## Abstract

Research on regulatory mechanisms in biological populations often focuses on environmental covariates. An integrated approach that combines environmental indices with organismal-level information can provide additional insight on regulatory mechanisms. Survival of spring/summer Snake River Chinook salmon (*Oncorhynchus tshawytscha*) is consistently low whereas some adjacent populations with similar life histories experience greater survival. It is not known if populations with differential survival respond similarly during early marine residence, a critical period in the life history. Ocean collections, genetic stock identification, and otolith analyses were combined to evaluate the growth-mortality and match-mismatch hypotheses during early marine residence of spring/summer Snake River Chinook salmon. Interannual variation in juvenile attributes, including size at marine entry and marine growth rate, was compared with estimates of survival and physical and biological metrics. Multiple linear regression and multi-model inference were used to evaluate the relative importance of biological and physical metrics in explaining interannual variation in survival. There was relatively weak support for the match-mismatch hypothesis and stronger evidence for the growth-mortality hypothesis. Marine growth and size at capture were strongly, positively related to survival, a finding similar to spring Chinook salmon from the Mid-Upper Columbia River. In hindcast models, basin-scale indices (Pacific Decadal Oscillation (PDO) and the North Pacific Gyre Oscillation (NPGO)) and biological indices (juvenile salmon catch-per-unit-effort (CPUE) and a copepod community index (CCI)) accounted for substantial and similar portions of variation in survival for juvenile emigration years 1998–2008 (R^2^>0.70). However, in forecast models for emigration years 2009–2011, there was an increasing discrepancy between predictions based on the PDO (50–448% of observed value) compared with those based on the NPGO (68–212%) or biological indices (CPUE and CCI: 83–172%). Overall, the PDO index was remarkably informative in earlier years but other basin-scale and biological indices provided more accurate indications of survival in recent years.

## Introduction

One focus of population ecology is the identification of environmental indices that are related to variation in population size or productivity [1], [2], [3]. Such relationships are often based on hypothesized mechanisms, such as a “stable ocean” [4] or “optimal upwelling window” [5], but the relationships fail to hold up over time [6], [7]. A parallel approach has been to identify a “critical period” in a species' life history, after which variation in the rate of mortality stabilizes [8], [9]. If the critical period is successfully identified, then the abundance or condition of a cohort during, or shortly after, this critical period should provide a robust indication of relative survival. This approach is not necessarily based on a mechanistic understanding of mortality but can focus research efforts by identifying the critical period(s) in a species' life history. Moreover, a combined approach can identify relevant local or regional environmental factors and also provide insight on the timing and mechanisms of major mortality events.

The Columbia River basin is the largest watershed on the west coast of the United States [10] and supports numerous populations of anadromous Chinook salmon, including five that are currently listed under the U. S. Endangered Species Act (ESA) [11]. Extensive modifications have been made to the river's hydropower system to minimize mortality of juvenile salmon during emigration to the ocean. Currently, in-river survival during migration averages 40–60% per year for populations that emigrate as yearlings after one year of freshwater rearing, although certain conditions, such as very low river flow, can result in mean annual in-river survival around 25% [12], [15]. However, overall survival to maturity remains relatively low (∼1%) for certain populations, such as spring/summer Chinook salmon, which return to their natal rivers in the spring and summer, from the Snake River, which is the largest tributary of the Columbia River. Other populations that also emigrate long distances (≥500 river kilometers, rkm), such as the spring Chinook salmon from the Mid-Columbia River, experience greater survival (∼3–4%) [12], [13]. These consistent differences in survival led to a hypothesis that multiple stressors during migration in the Snake River increase juvenile mortality during estuarine and early ocean residence, a phenomenon referred to as “delayed mortality” [14], [15]. However, it is not yet clear if yearling emigrants from the Snake and Mid-Upper Columbia River, which emigrate at comparable times and sizes, respond similarly during early marine residence, which is considered a critical period for anadromous salmonids [16], [17].

Columbia and Snake River Chinook salmon that emigrate as yearlings enter the northern California Current, an eastern boundary current characterized by seasonal upwelling. For the Mid-Upper Columbia River spring Chinook salmon genetic stock group, there is evidence in support of the “growth-mortality hypothesis”, which postulates that larger, faster growing individuals survive better due to their enhanced ability to capture prey and avoid predation compared with smaller counterparts [18], [19], during their early marine residence. Tomaro et al. [20] determined that interannual variation in early marine growth and juvenile size after ∼30 d in the coastal ocean was highly correlated with subsequent returns of Mid-Upper Columbia River spring Chinook salmon. However, there was weaker support for the “match-mismatch hypothesis”, which posits that survival during early life is related to an appropriate overlap between predators and their prey [21], [22].

For spring/summer (sp/su) Snake River Chinook salmon, which also emigrate as yearlings, prior research indicates that in-river juvenile survival is positively related to body size [23], [24] and that earlier emigrants tend to survive at higher rates than later emigrants [25]. However, information on mechanisms of mortality during early marine residence is more limited and there is a lack of information on the relative importance of freshwater, estuarine, and marine factors on the survival of Snake River sp/su Chinook salmon.

We combined ocean collections of juvenile salmon with genetic stock identification and otolith structural and chemical analyses to evaluate the growth-mortality and the match-mismatch hypotheses during initial marine residence in Snake River sp/su Chinook salmon. We also evaluated the ability of juvenile attributes and environmental indices during early marine residence to account for interannual variation in survival. First, we characterized interannual variation in juvenile salmon attributes, such as size at marine entry and marine growth rate, and compared these attributes with estimates of survival. Second, we evaluated the relative support for the growth-mortality and match-mismatch hypotheses during early marine residence by comparing environmental variables, juvenile attributes, and survival. Third, we determined which environmental variables accounted for the observed interannual variation in juvenile attributes, specifically mass at capture. Finally, we used multiple linear regression and multi-model inference to evaluate the relative importance of biological and physical indices in describing interannual variation in survival. We used a retrospective approach to hindcast survival for years in which we had ocean survey data (1998–2008) and a forecast approach where we used the top hindcast models to predict survival during recent emigration years (2009–2011).

## Materials and Methods

### Study Organism

Snake River sp/su Chinook salmon was listed as “threatened” under the ESA in 1992. The current management unit, identified as an Evolutionarily Significant Unit (ESU), includes all naturally spawned sp/su Chinook salmon in the mainstem Snake River and the Tucannon, Grande Ronde, Imnaha, and Salmon river sub-basins as well as numerous artificial propagation units [26]. An estimated population of >1,000,000 fish in the late 1800s declined to <5,000 in the 1990s [26]. Survival of Snake River sp/su Chinook salmon, as indicated by smolt-to-adult return ratios (SARs) which provide a measure of overall survival from the emigrating smolt stage to the returning adult stage, ranged from <0.005 to 0.032 (or <0.5 to 3.2%) from 1998 to 2008 (http://www.fpc.org/).

**Figure 1:**
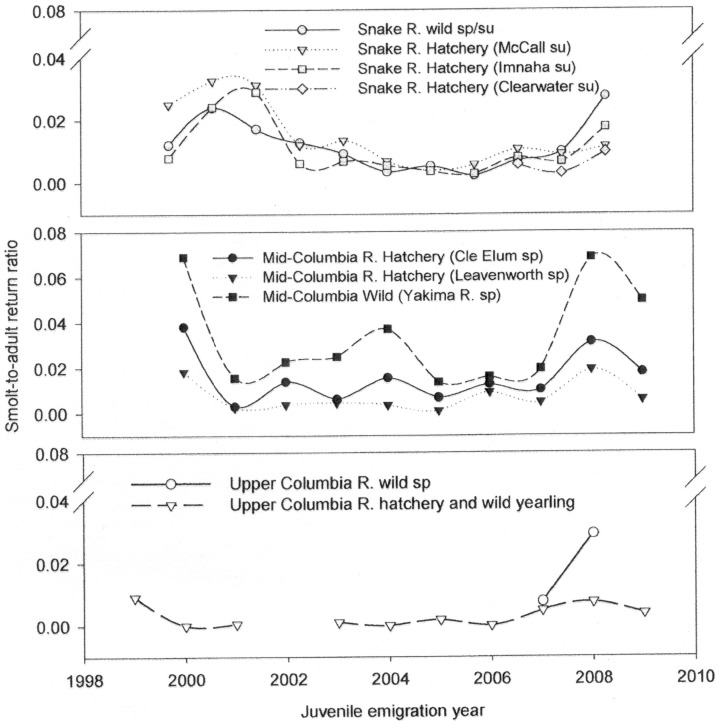
Smolt-to-adult return ratios (SARs). Estimates of SARs for populations of spring and summer Chinook salmon from the (a) Snake River, (b) Mid-Columbia River, and (c) Upper Columbia River. SARs are presented as percentages without the inclusion of precocious males (jacks) and were acquired from the Fish Passage Center (http://www.fpc.org/). The run timing (sp  =  spring, su  =  summer) and the hatchery or river of origin are also included.

We used SARs for Snake River sp/su Chinook salmon as the survival metric for comparison with juvenile attributes and environmental indices. Snake River sp/su Chinook salmon emigrate as yearlings and the majority (>80%) spend two years in the ocean prior to returning to spawn [26]. SAR estimates include all ages-at-maturity for a particular brood year. We used the SARs that were based on detections of emigrating smolts and returning adults with Passive Integrated Transponder (PIT) tags at Lower Granite Dam (LGD) on the Snake River (http://www.fpc.org/). We used the composite estimate for wild Snake River sp/su Chinook salmon without jacks, which are precocious males that return after one year in the ocean, in subsequent analyses [12] (Fig. 1). We selected this metric because: (1) all SAR estimates for this population were highly correlated from 1998–2008 (r = 0.986 for SARs with and without jack; r = 0.740 for SARs for wild and hatchery composite; and r = 0.978 for SARs from LGD to Bonneville Dam and from LGD-LGD; (2) the available estimates for the proportion of jacks within a brood year were not significantly correlated with SARs (r = 0.455, 2000–2010); and (3) we were interested in making inference about the survival of the naturally-spawned (presumably wild) portion of the population, which is of primary conservation concern.

### Juvenile collection and genetic stock identification

**Figure 2:**
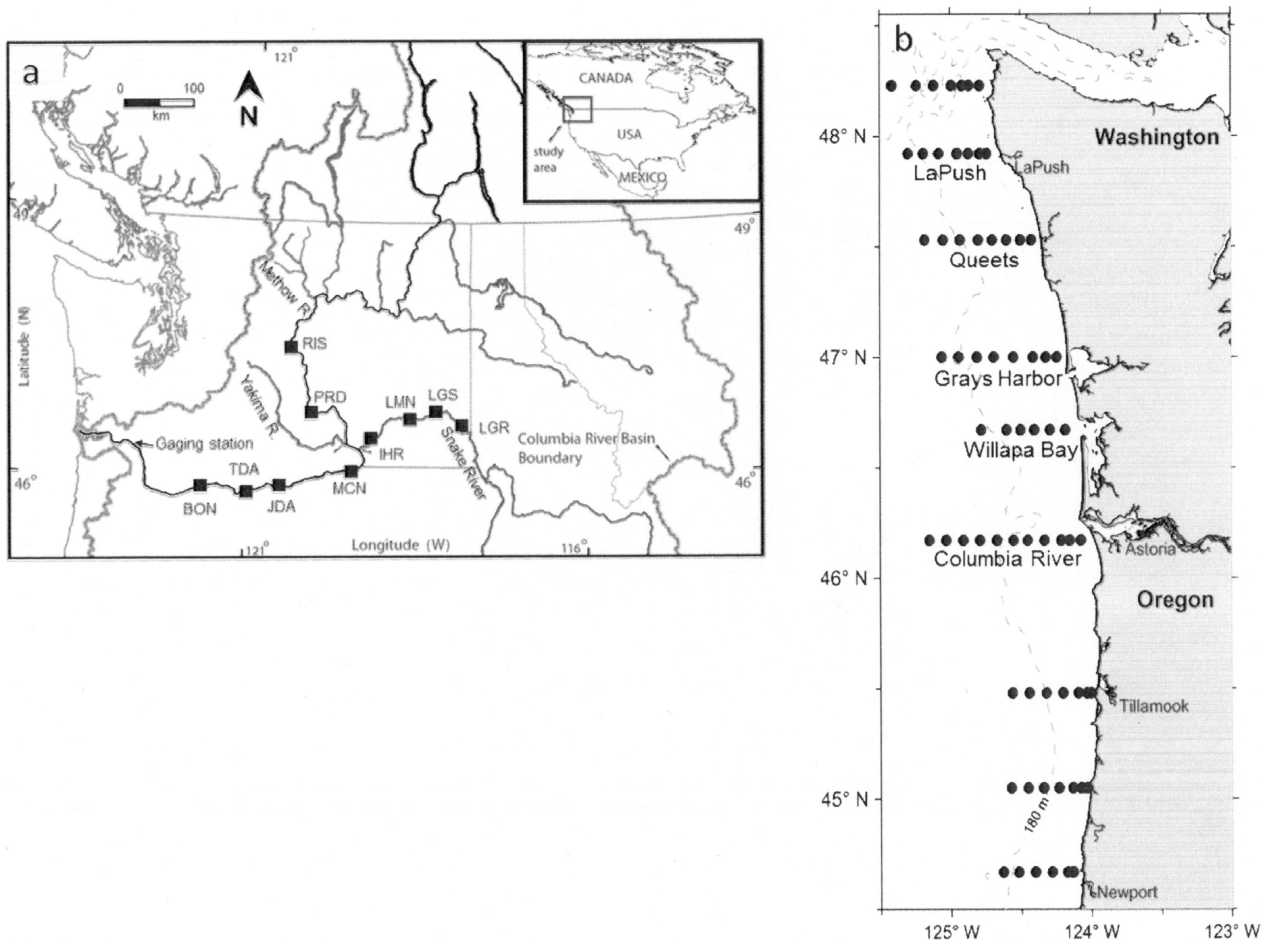
Map of study location. (a) Columbia River watershed with locations of the mainstem dams and gaging station referred to in text. BON  =  Bonneville Dam; TDA  =  The Dalles Dam; JDJ  =  John Day Dam; MCN  =  McNary Dam; ICH  =  Ice Harbor Dam; LMJ  =  Lower Monumental Dam; LGS  =  Little Goose Dam; LGR  =  Lower Granite Dam. (b) Transect and station locations for ocean collections used in this study.

Surveys occurred off the coast of Washington and Oregon during late May and late June from 1998 to 2008 (Fig. 2). A Nordic 264 rope trawl was towed in surface waters, and catches were standardized to density (catch-per-unit-effort, CPUE) of yearling Chinook salmon based on trawl width and the distance towed (fish km^−1^). On board, fish were placed immediately on ice, identified, measured (fork length (FL), mm), and sacrificed (i.e., frozen). All animal work was conducted according to relevant national guidelines. Fish were collected under ESA Section 10 permit #1410–7A, which is the federal process for research conducted by the National Oceanic and Atmospheric Administration (NOAA) that involves ESA-listed species. During this study, NOAA collections of fishes did not undergo a separate review by an Institutional Animal Care and Use Committee.

In the laboratory, fish were re-measured and weighed (±0.1 g) and fin clips and otoliths were collected. Fin clips were used to genotype juveniles at 13 microsatellite DNA loci [27] and assign individuals to stock group using a standardized genetic database [28], [29]. Stock assignments were made with the program ONCOR [30] and the likelihood model of Rannala and Mountain [31]. For Columbia River Chinook salmon, the stocks that are identifiable using the standardized set of microsatellite loci are mostly concordant with the eight ESUs in the basin [27], [29]. However, translocations of hatchery populations in the region have been very extensive [26] and in recent genetic studies, the Mid and Upper Columbia River spring-run ESUs have been combined into a single stock [20], [27], [29]. From 1998 to 2008, 755 individuals from the coastal surveys were classified to the Snake River sp/su Chinook salmon genetic stock group. However, the years 1998, 2001, and 2005 were not included in the analysis of juvenile attributes due to low CPUE and therefore small sample size (n<10). Therefore, 732 juveniles collected in 1999–2000, 2002–2004, 2006–2008 were included in subsequent analyses.

### Juvenile migratory attributes: size at and timing of marine entry and early marine growth

A subsample of the juveniles was selected for otolith structural and chemical analyses to determine size at, and timing of, marine entry as well as marine growth and migration rates. The temporal (May vs. June) and spatial (across transects) (Fig. 2) distribution of the subsample was similar to the overall catch (χ^2^<27.5, p>0.05 for all years except 2006–2008). In the 2007 and 2008 subsample, there was a slight bias towards juveniles collected along the Columbia River transect in May (27.5<χ^2^<29.5). In 2006, there was an over-representation of juveniles from a northern transect (LaPush) in May and an under-representation in June (Queets River) (χ^2^ = 121.8). Overall, however, we consider the subsample representative of the ocean catch of juvenile Snake River sp/su Chinook salmon.

Sagittal otoliths were removed, cleaned, and polished using wet-or-dry paper (240–2500 grit) and lapping film (1–30 µm) to expose the dorsal-ventral growth axis using standard procedures for elemental analysis [32]. Otolith Sr and Ca were measured along the dorsal-ventral growth axis using a VG PQ ExCell inductively coupled plasma mass spectrometer with a New Wave DUV193 excimer laser. The laser was set at a pulse rate of 7 Hz and travelled across the sample at 5 µm s^−1^ with a spot size of 30 or 50 µm. Normalized ion ratios were converted to molar ratios using standard procedures [33], [34]. Instrument precision (mean percent relative standard deviation) was 4.5% for Ca and 4.7% for Sr across all samples and days (n = 50) and accuracy for Sr:Ca was 4% (n = 5) based on USGS MACS-1.

Image analysis was combined with Sr:Ca data to determine otolith width (OW) at marine entry and to estimate the date of marine entry [35]. For each individual, the OW at the time of marine entry was determined by the initial and abrupt increase in otolith Sr:Ca, which indicates exit from freshwaters, prior to stabilizing at marine values [36], [37]. We enumerated the increments deposited after the initial and abrupt increase in otolith Sr:Ca to determine residence in brackish/ocean (hereafter referred to as “marine”) waters. To determine date of marine entry, the duration of marine residence was subtracted from the date of capture. Marine migration distance was conservatively estimated as the linear distance between the mouth of the Columbia River (N 46.253°, W 124.059°) and the capture station plus 32.1 km to account for travel through the estuary. We divided the migration distance (km) by the marine residence time (d) to calculate the mean migration rate (km d^−1^) for each fish, which was converted to body lengths per second (bl s^−1^) based on estimated size at marine entry.

We developed a direct back-calculation model based on the fork length to otolith width relationship of yearling sp/su Chinook salmon from the interior Columbia River basin that were collected from 1999–2008 (r^2^ = 0.82, n = 362, p<0.001). We determined size at marine entry using Eq. (1). 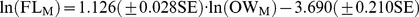
(1)where FL_M_  =  fork length (mm) at marine entry, and OW_M_  =  otolith width (µm) at marine entry. Marine growth rates (% d^−1^, mm) were then determined by subtracting estimated size at marine entry from size at capture, dividing by size at marine entry, and multiplying by 100.

### River, estuary, and ocean indices

We compiled indicators of river, estuary, and ocean conditions during juvenile emigration for comparison with juvenile attributes and survival. Data on daily discharge in the lower river were obtained from the United States Geological Survey (Site 14246900 at 46°N, 123°W). We characterized attributes of the Columbia River plume, defined using a cutoff salinity of 28, with the output of simulation databases (www.stccmop.org/datamart/virtualcolumbiariver) [38], including plume size (area of the plume surface and volume of the 3D plume) and location (expressed in terms of coordinates of the centroid of the surface plume) [39], [40]. We hypothesized that conditions during emigration would be the most relevant to survival variation but physical indices were averaged across seasons (January to March, April to June, etc.) to identify the most appropriate period.

We examined two basin-scale environmental indices, the Pacific Decadal Oscillation (PDO) and the North Pacific Gyre Oscillation (NPGO), which are statistically independent modes of variation in ocean sea surface temperature (SST) and sea level height (SLH), respectively. The PDO is defined as the leading principal component of North Pacific monthly SST variability poleward of 20°N [41]. Negative values of the PDO indicate cooler SST and relatively high salmon production off the west coast of North America [42], [43]. The NPGO is the second leading principal component of variability in North Pacific SLH and is correlated with salinity, nutrients, and chlorophyll values [44]. Monthly mean values for these indices were downloaded from http://jisao.washington.edu/pdo/PDO.latest and www.o3d.org/npgo/data/NPGO.txt. Physical indices were averaged across seasons (January to March, April to June, etc.) to identify the most appropriate periods.

To provide an indication of interannual variation in ocean productivity, we also examined the Copepod Community Index (CCI). The CCI is a numerical representation of all copepod species that are present in more than 5% of the samples collected biweekly 9 km offshore of Newport, Oregon using a 50-cm diameter, 202-µm mesh ring net towed vertically from 5 m above the sea floor to the surface. The values are rotated Axis 1 scores of a non-metric multidimensional scaling ordination of species abundance by sample date from 1996 to 2010 [45], [46]. During spring and summer, negative CCI values indicate the presence of a “northern community”, i.e., boreal, neritic taxa that are large and lipid-rich, whereas positive values indicate the presence of an “offshore or southern community” comprised of smaller, relatively lipid-poor species [47]. The CCI may be indicative of the nutritional quality of the food web supporting juvenile salmon and their prey.

In the northern California Current, there is a marked spring transition with the initiation of seasonal upwelling [48]. The physical spring transition is defined as the first day that the 10-d average for upwelling indices is positive and sea level height is negative [49]. The biological spring transition is defined as the first day that the copepod community present offshore of Newport, Oregon is dominated by lipid-rich, boreal species [45], [50].

### Evaluation of the Match-Mismatch and Growth-Mortality Hypotheses

The majority of juvenile salmon along the west coast of the US enter marine waters during the productive spring/summer upwelling season [51]. Hence, both the timing and magnitude of productivity during the upwelling season could be important to the growth and survival of juvenile salmon. There is evidence that, within years, juveniles survive better when they emigrate earlier in the spring [25]. However, it is not clear if interannual variation in survival is related to timing of in-river transit or marine entry, and there may be an interaction between the timing of marine entry and the initiation of the upwelling season. Therefore, in addition to our examination of river, estuary, and ocean conditions during juvenile emigration, we also estimated the date of marine entry in relation to the physical and biological transitions in coastal waters. For each individual, we determined the number of days between their marine entry and the physical (ME_PT_) and biological spring (ME_BT_) transition dates for that emigration year (marine entry - ME_PT_ or ME_BT_) and generated annual mean estimates for comparison with survival. The expectation is that if the timing of marine entry of those juveniles that survived initial marine residence was important for subsequent survival, there would be significant, positive relationships between SARs and ME_PT_ O ME_BT_.

In Pacific salmon and other fishes, survival is often positively related to body size at some point in the early life history [18], [19], [24]. However, it is not clear when the size-survival advantage occurs, e.g., during in-river migration or early marine residence. By determining size at marine entry and early marine growth rates, we can examine the relative importance of interannual variation in entry size and early marine growth on survival. Therefore, we related SARs to juvenile size at marine entry, early marine growth, and size at capture using correlation analyses (1999–2000, 2002–2004, 2006–2008).

For all correlations, we adjusted degrees of freedom when determining significance for Pearson product-moment correlation coefficients (^†^P) if needed to account for auto-correlation within time series, as recommended by Pyper and Peterman [52]. Given the high rates of marking Snake River Chinook salmon hatchery fish by clipping adipose fins and injecting coded wire tags across the study period (>90%; Regional Mark Information System, www.rmpc.org), we also compared attributes of marked and unmarked fish to determine if there were significant differences between these two groups.

### Hindcasting survival

Our intention was to evaluate the relative importance of local and regional indices in describing interannual variation in survival as well as to determine if juvenile attributes after a hypothesized critical period provide additional or unique information on survival variation (1999–2000, 2002–2004, 2006–2008). First, for the years with adequate numbers of juveniles, we compared SARs with size at, and timing of, marine entry, marine growth rate, and size at capture to evaluate their relative importance in relation to survival using correlation analyses. Second, we used a multiple linear regression approach to determine which environmental indices accounted for variation in those juvenile attributes that were found to be related to survival (SARs) in our correlation analyses. Third, we evaluated the relative ability of the environmental indices to hindcast survival directly, including all years (1998–2008). Variables for model inclusion were selected based on visual inspection and correlation analysis and transformed when necessary to meet parametric assumptions. Variables with cross-correlations >0.50 were not included in the same model. We calculated Akaike Information Criteria adjusted for small sample size (AIC_C_) to evaluate models [53]. Normalized likelihood values (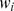) were calculated using the following: 
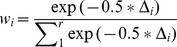
where 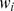 are Akaike weights for model 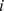, the numerator is the likelihood for model 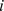, and the denominator is the sum of the relative likelihoods for models 1 to *r.* 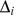
represents the difference between the AIC_c_ of the best model and the others. We also calculated model-averaged parameter coefficients and variance using standardized coefficients to evaluate the relative importance of variables in the models using the following:



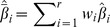


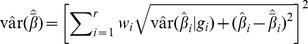
where 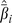 denotes the model-averaged coefficient, 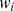 is the model weight, and 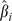 is the coefficient for model 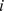. The unconditional variance estimate 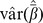 was determined using the weight 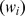 and variance (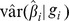) of each model [53].

## Results

### Juvenile migratory patterns

**Figure 3:**
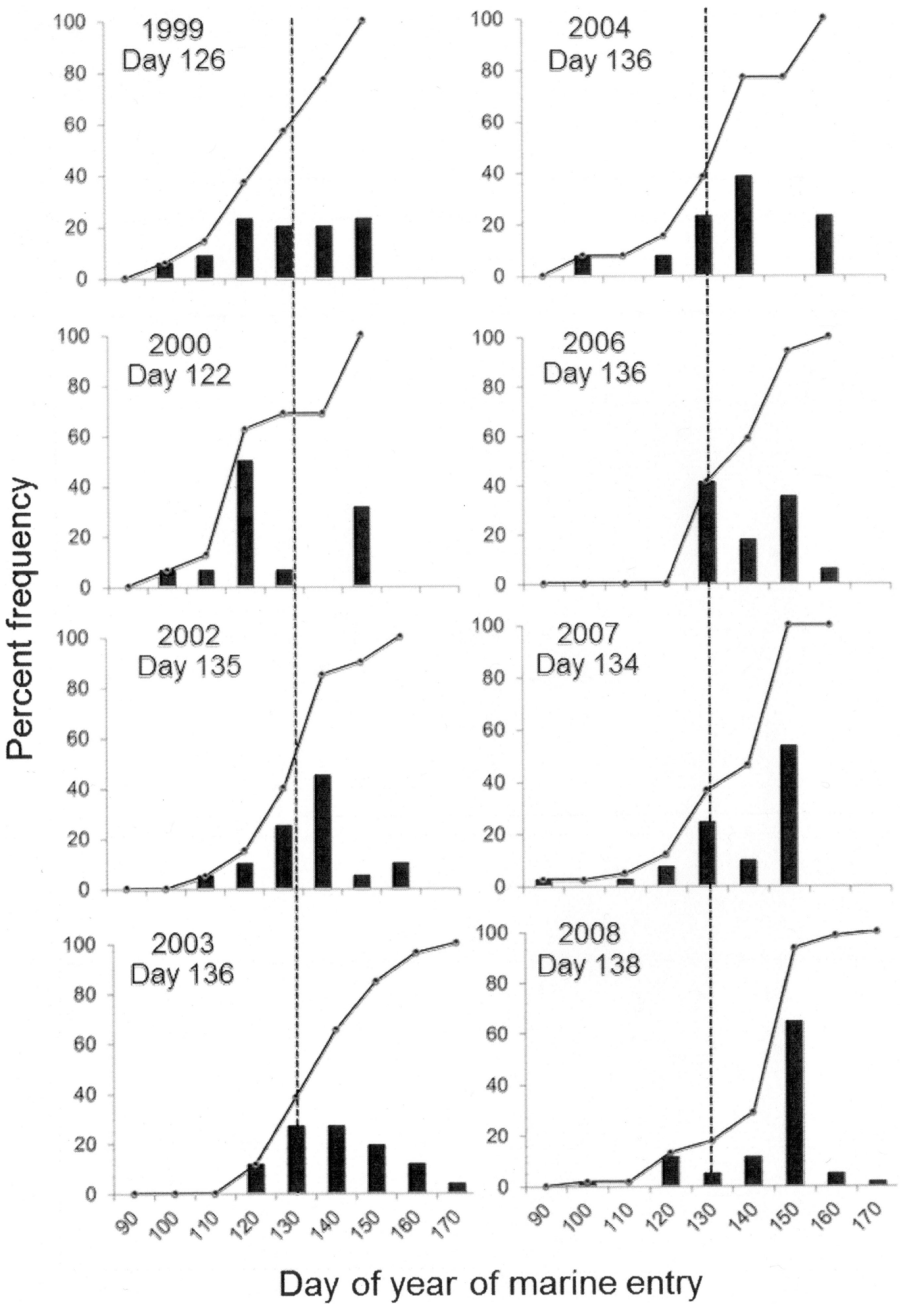
Timing of marine entry for juvenile Snake River sp/su Chinook salmon. Percent frequency for the day of year of marine entry (bars) as estimated from otolith chemical and structural analyses (n = 230). Year and mean day of year of emigration are included on each graph. Black lines represent cumulative frequency. Dotted lines represent overall mean date of emigration (May 12 = 133).

Across all years, the annual mean emigration date occurred from 6 May to 18 May and all juveniles emigrated between 20 April and 19 June with a shift toward later emigration in 2006–2008 (Fig. 3). The mean proportion of marked, and presumably hatchery, fish in ocean collections was 80.5% and ranged from 67% in 2000 to 89% in 2004 and 2006 (Table 1). Fourteen of these marked fish had PIT tags [54], which provided data on juvenile size at release and information on when and where they were detected within the Columbia River hydropower system. These tagged fish provided an opportunity to compare our otolith-derived estimates for size at, and timing of, marine entry with available information (http://www.ptagis.org/) (Table S1 in File S1). Two fish (14%) were estimated to be smaller at marine entry than at release (by ∼6%) and these were transported by barge through the hydropower system quickly (≤10 d). Additionally, all of our estimates of marine entry were within five days of the fish's last detection within the hydropower system. Overall, these data provide a qualitative indication of the ability to estimate size at, and timing of, emigration.

**Table 1:** Biological characteristics of Snake River sp/su Chinook salmon.

Year	Size at marine entry (FL, mm)	Emigration day	Marine growth (% d^-1^ mm)	*n*	Size at capture (FL, mm)	*n*	Proportion marked: ocean	Hatchery releases (10^6^)	Proportion marked: hatchery	SAR	Mid/Upper CR Hatchery releases (10^6^)
1999	130.7 (3.6)	126 (2.4)	0.70 (0.06)	28	160.5 (3.8)	169	0.72	8.68	0.96	0.024	5.5/2.8
2000	134.0 (3.6)	123 (3.6)	0.83 (0.10)	12	169.3 (5.8)	43	0.67	7.31	0.93	0.017	6.0/3.3
2002	150.2 (5.6)	135 (3.9)	0.47 (0.13)	11	158.8 (6.3)	35	0.80	11.93	0.90	0.009	5.5/3.2
2003	132.0 (4.9)	136 (2.7)	0.65 (0.05)	19	152.2 (4.1)	55	0.73	12.30	0.94	0.003	5.4/2.5
2004	137.0 (7.3)	136 (4.8)	0.52 (.012)	10	150.4 (8.3)	27	0.89	12.55	0.99	0.005	5.4/2.7
2006	126.7 (4.6)	136 (2.7)	0.58 (.09)	12	146.6 (4.9)	101	0.89	11.88	1.00	0.007	5.6/3.0
2007	139.6 (3.0)	135 (2.0)	0.57 (.05)	23	151.4 (3.0)	102	0.86	11.05	0.97	0.010	5.2/3.0
2008	129.1 (3.0)	139 (1.7)	0.81 (.05)	36	157.0 (3.3)	199	0.88	10.79	0.96	0.027	5.6/2.9

Mean (SE) size at, and date of, marine entry, growth rate, and size at capture by emigration year for juvenile spring/summer Chinook salmon from the Snake River. The proportion of fish in ocean collections that were marked with adipose fin clip or other tag and the estimated number of spring/summer juvenile Chinook salmon released in the Snake River basin each year are presented. The majority of hatchery production occurs at Dworshak National Fish Hatchery and the McCall, Clearwater, and Rapid River hatcheries. The smolt-to-adult return ratio (SAR) for each emigration year is also included. Hatchery releases for Mid and Upper Columbia River spring Chinook are included for reference (Mid/Upper CR).

**Figure 4:**
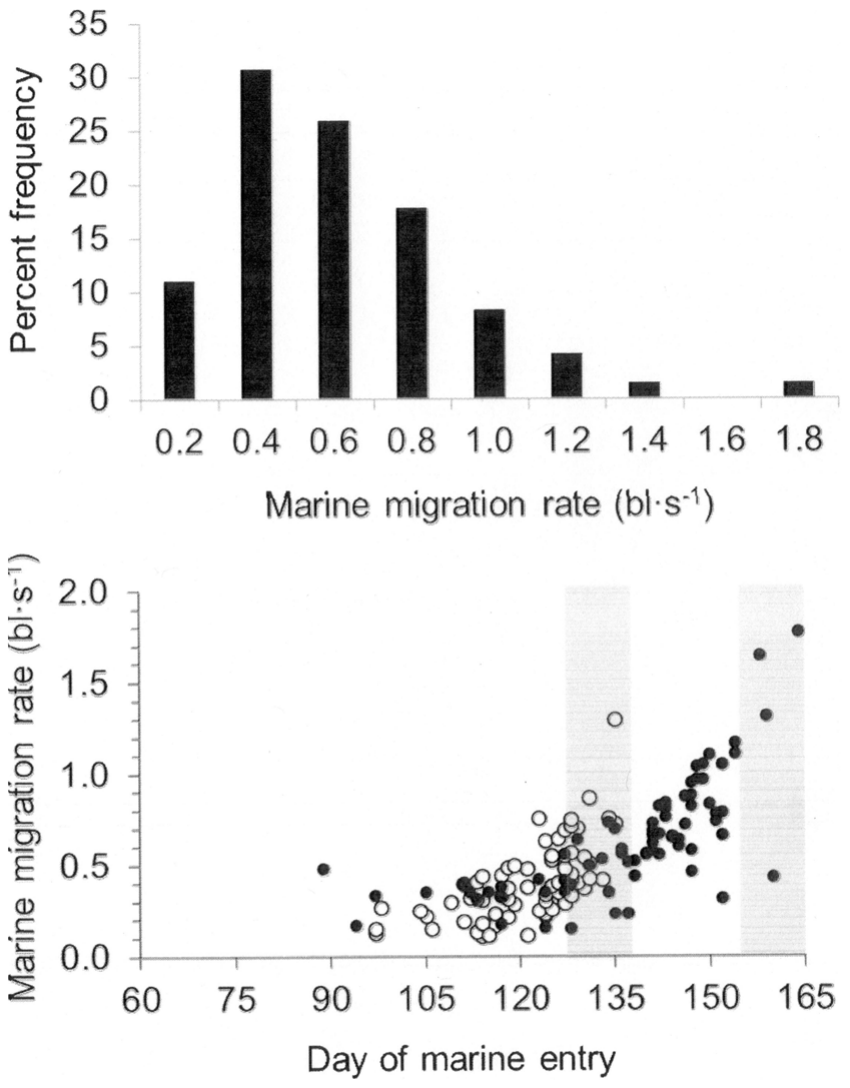
Juvenile Snake River sp/su Chinook salmon marine migration rates. (a) Estimated marine migration rates (bl·s^−1^) for juvenile Snake River sp/su Chinook salmon across all years. (b) Individual marine migration rates for all years (1999–2000, 2002–2004, 2006–2008). Filled circles represent juveniles collected during May cruises (n = 70) and open circles represent juveniles collected during June cruises (n = 77). Shaded boxes indicate cruise dates.

A relatively high percentage (34%) of the juveniles displayed no evidence of marine residence, i.e., no elevated Sr:Ca in their otoliths, which indicates recent marine entry (<5 d) [55], [56]. Therefore, individual residence in coastal waters prior to capture ranged from 1 to 81 d with an overall mean of 20 d (±17.9 SD). For those individuals with evidence of marine residence in their otoliths, mean annual marine growth rate ranged from 0.47% d^−1^ in 2002 to 0.83% d^−1^ in 2000 (Table 1). Mean migration rate ranged from 0.11 to 1.77 bl s^−1^ and tended to increase later in the year (Fig. 4) (mean  = 0.40 bl s^−1^±0.21 SD and 0.64 bl s^−1^±0.32 SD in May and June, respectively, t-test, P<0.05).

There were significant interactions between year and presumptive origin (marked vs. unmarked) for most juvenile attributes, including size at marine entry rate (F_7,130_>2.7, P<0.05), marine migration rate (F_7,130_>2.4, P<0.05), marine growth rate (F_7,130_>2.4, P<0.05) and size at capture (F_7,715_ = 2.6, P<0.001) (Table S2 in File S1). There was no interaction between origin and year for the date of marine entry, and unmarked fish entered an average of 2 days later than marked fish (F_1,7_ = 7.0, P<0.05). We observed no other consistent differences between origins.

### Match-Mismatch and Growth-Mortality Hypotheses

**Figure 5:**
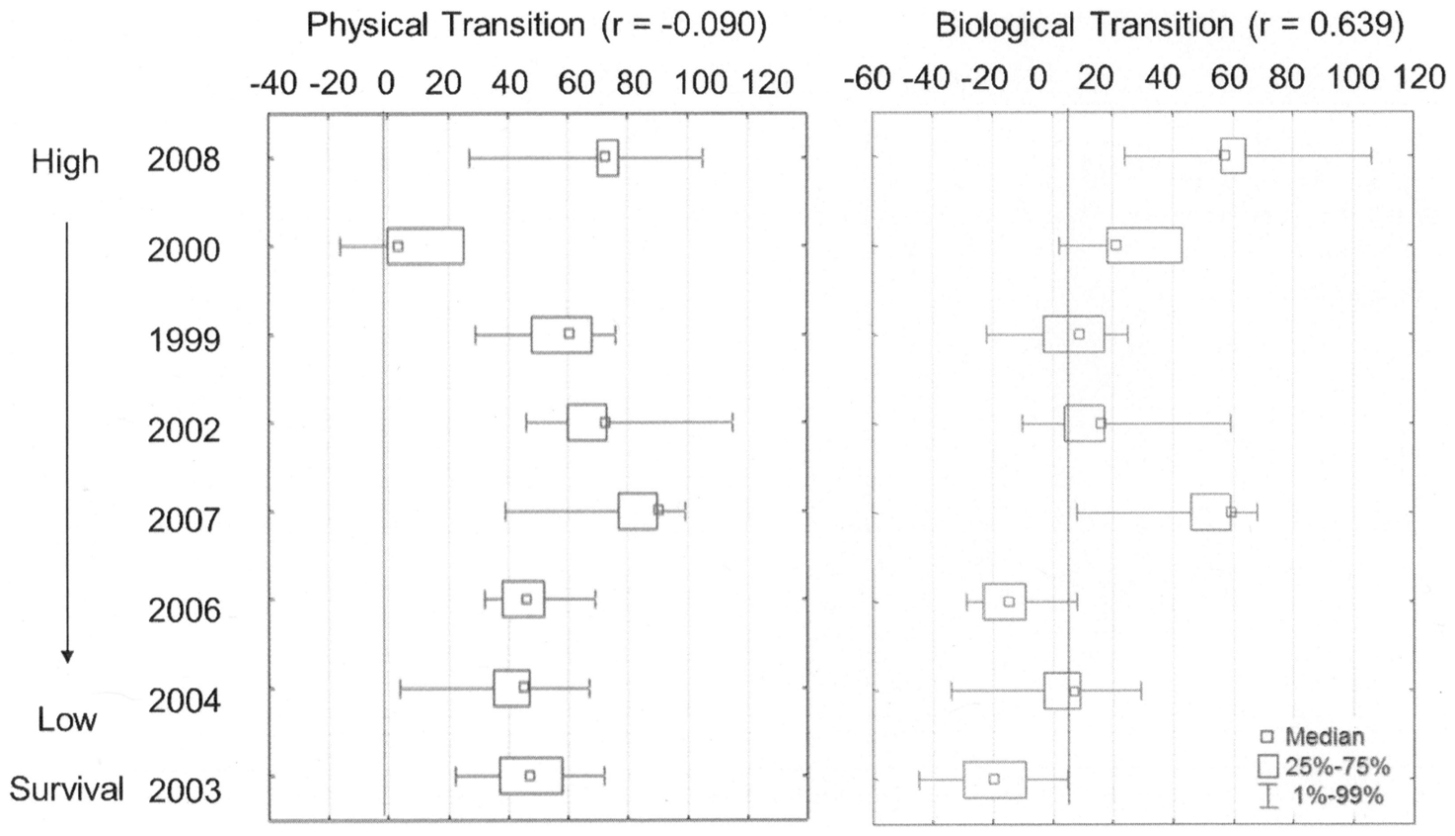
Timing of marine entry for Snake River sp/su Chinook salmon in relation to ocean conditions. Mean date of marine entry in relation to the (a) physical and (b) biological spring transition. Years are ranked from the highest to lowest adult returns and labeled by juvenile emigration year.

We observed minimal evidence in support of the match-mismatch hypothesis (Table 2). Juveniles consistently entered marine waters after the physical spring transition but there was no statistically significant relationship with the physical or biological transition (Fig. 5). However, there was a non-significant positive trend (r = 0.639) with higher SARs when fish emigrated later in the year relative to the biological transition (Fig. 5).

**Table 2:** Pearson's correlation coefficients for comparisons between biological and physical indices and smolt-to-adult return ratios (SAR).

	FL (mm)	M (g)	SAR
1. May marine density (yearling km^−1^)	**0.759**	**0.773**	**0.770**
2. June marine density (yearling km^−1^)	**0.797**	**0.833**	**0.886**
3. Marine growth rate (% d^−1^, mm)	**0.778**	**0.805**	**0.727**
4. Size at marine entry	−0.100	−0.142	−0.252
5. Juvenile migration rate (bl sec^−1^)	−0.315	−0.253	−0.070
6. Columbia River flow_4___7_ (m^3^ s^−1^)	0.311	0.310	0.514
7. ME_BT_	0.510	0.547	0.639
8. ME_PT_	−0.157	−0.129	0.090
9. PDO_7___9_	**−0.853**	**−0.874**	**−0.918**
10. NPGO_4___6_	**0.906**	**0.893**	**0.715**
11. Plume area_4___7_ (km^2^)	**0.894**	**0.901**	**0.935**
12. CCI_6_	**−0.868**	**−0.860**	**−0.799**
13. SAR	**0.883**	**0.902**	1.000

FL  =  fork length (mm). M =  mass (g). ME_BT_ is the annual mean day of marine entry in relation to the biological spring transition whereas ME_PT_ is the annual mean day of marine entry in relation to the physical spring transition (see text for additional details). Subscripts indicate the months over which data were averaged (e.g., PDO_7___9_ =  mean PDO from July to September). Adjusted significant values (^†^P<0.05) are indicated by bold letters. n = 8 for all comparisons. Variables were ln-transformed (FL, M, and (4), (6), (7) and (12)) or square root transformed (SAR and (2)) to normalize distributions and homogenize variances. Years included are 1999, 2000, 2002–2004, 2006–2008.

**Figure 6:**
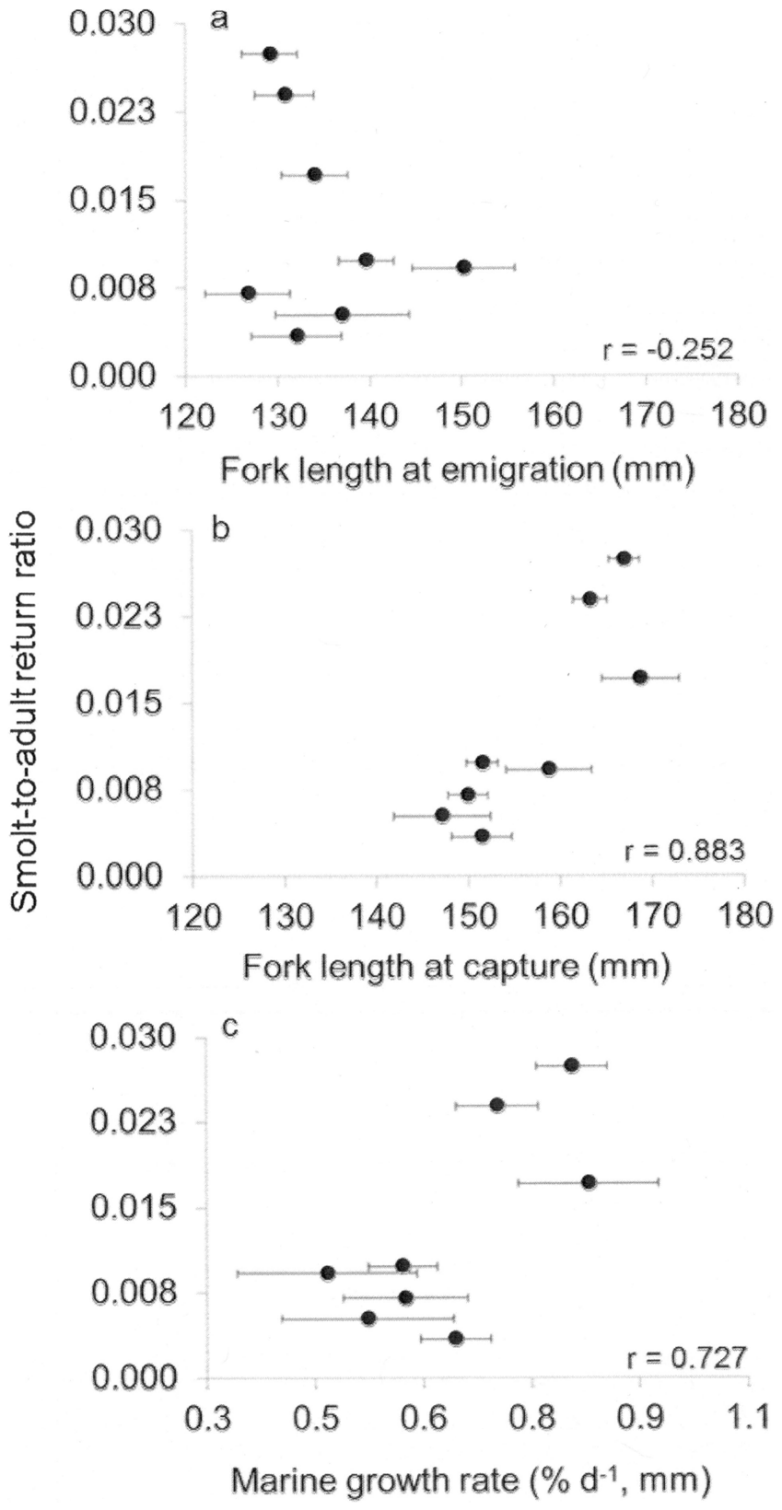
Relationships between survival and juvenile salmon attributes. Relationship between smolt-to-adult return ratios (SAR) and mean annual size and growth characteristics for Snake River sp/su Chinook salmon. Back-transformed SAR versus mean (±SE) (a) juvenile size at marine entry, (b) size at capture, (c) and marine growth rate.

There was stronger support for the growth-mortality hypothesis after marine entry (Table 2, Fig. 6). Mean size at marine entry displayed negative, non-significant trends with survival but length (Fig. 6b) and growth (Fig. 6c), measured an average of 20 d after marine entry, were strongly, positively related to survival (Fig. 6c, r>0.73).

Given that fish mass at capture was the most informative juvenile attribute in relation to survival (Table 2), we evaluated the ability of physical and biological variables to hindcast interannual variation in juvenile size. This analysis could only be completed for years with adequate ocean collections of juveniles (1999–2000, 2002–2004, 2006–2008). Columbia River plume area and volume are highly correlated (r = 0.963); therefore, we used plume area in our models due to its slightly better relationship with juvenile mass at capture (r = 0.901 vs 0.871). The top five out of 15 possible models accounted for similar proportions of the variance in juvenile mass at capture (≥0.74) (Table 3). In general, yearlings were heavier in years in which the plume was larger, the PDO index was more negative, the NPGO index was more positive, and the CCI was more negative, i.e., dominated by northern, boreal copepod species (Table 2). Given the family of models, the model that incorporated plume area during emigration (April through July) was 1.4 to 2.5 times more likely than models based on basin-scale indices (NPGO_4___6_ and PDO_7___9_) and >3.5 times more likely than the models with the CCI_6_. As all four of these variables were correlated (r≥0.59) and thus not included in the same model, we compared the model-averaged coefficients, which indicated that PlArea_4___7_ was the most informative variable 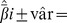
0.359±0.086), followed by NPGO_4___6_ (0.260±0.057), PDO_7___9_ (−0.141±0.022), and the CCI_6_ (−0.041±0.042).

**Table 3:** Model comparisons for juvenile mass.

Model	RSS	AIC_c_	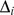	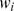	R^2^
PlArea_4___7_	0.050	−28.672	0.000	0.398	0.812
NPGO_4___6_	0.054	−28.049	0.623	0.291	0.797
PDO_7___9_	0.062	−26.860	1.812	0.161	0.765
CCI_6_	0.069	−26.067	2.605	0.108	0.740
CCI_6,_ (CCI_6_)^2^	0.029	−23.589	5.083	0.031	0.890

Results for models describing variation in juvenile fish mass (g) after initial marine residence for Snake River spring/summer Chinook salmon based on ocean conditions during juvenile emigration. CCI_6_ =  Copepod Community Index in June; PlArea_4___7_ =  mean plume area from April to July; NPGO_4___6_ =  mean value from April to June; PDO_7___9_ =  mean value from July to September, RSS =  residual sum of squares, AIC_c_  =  Akaike Information Criteria adjusted for small sample size. 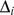 represents the difference between the AIC_c_ of the best model and the others. 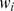 indicates the relative likelihood of the model given the data. Variables were transformed (logarithm or square root) to normalize distributions and homogenize variances.

### Local and regional indices and survival

Given the relative importance of plume area in accounting for variation in juvenile mass at capture, plume area was included in our initial comparison of models to hindcast SARs, which limited the analysis to 1999–2008 because no plume simulations are available for 1998. However, plume size was not included in the top ten models, therefore model comparisons were completed without plume metrics across emigration years 1998 to 2008. Interannual variation in SARs was relatively well-described (R^2^>0.70) by physical and biological conditions during emigration. In general, SARs were higher when the PDO_7___9_ and the CCI_6_ were more negative, the NPGO_4___6_ was more positive, and CPUE_6_ was greater. The two top hindcast models included the PDO_7___9_, and they both were >6.7 times more likely given the data than any model that included CPUE_6_ (Table 4). PDO_7___9_ was the most informative variable (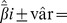
−0.692±0.217), followed by CCI_6_ (−0.115±0.039), CPUE_6_ (0.059±0.007), and the NPGO_4___6_ (0.023±0.002).

**Table 4:** Model results for salmon survival.

Model	RSS	AIC_c_	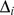	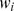	R^2^
1. PDO_7___9_, CCI_6_	0.0024	−78.148	0.000	0.430	0.830
2. PDO_7___9_	0.0039	−78.054	0.095	0.410	0.724
3. CPUE_6_	0.0055	−74.235	3.913	0.061	0.731
4. PDO_7___9_, (PDO_7___9_)^2^	0.0035	−73.802	4.346	0.049	0.748
5. NPGO_4___6_, CPUE_6_	0.0041	−72.163	5.985	0.022	0.707
6. NPGO_4___6_	0.0067	−71.953	6.196	0.019	0.520
7. CCI_6_	0.0088	−69.014	9.134	0.004	0.373
8. CCI_6_, (CCI_6_)^2^	0.0055	−68.872	9.276	0.004	0.507
9. CRFlow_4___7_	0.0121	−65.495	12.653	0.001	0.135

Comparison of models describing variation in survival of Snake River sp/su Chinook salmon based on conditions during juvenile emigration (1998 to 2008). PDO_7___9_ =  mean value from July to September; CCI_6_ =  Copepod Community Index in June; CPUE_6_ =  catch of yearling Chinook (fish km^−1^) in June; NPGO_4___6_ =  mean value from April to June; and CRFlow_4___7_ =  Columbia River flow from April to July. RSS  =  residual sum of squares, AIC_c_  =  Akaike Information Criteria adjusted for small sample size. 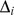 represents the difference between the AIC_c_ of the best model and the others. 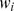 indicates the relative likelihood of the model given the data. Variables were transformed (logarithm or square root) to normalize distributions and homogenize variances.

**Figure 7:**
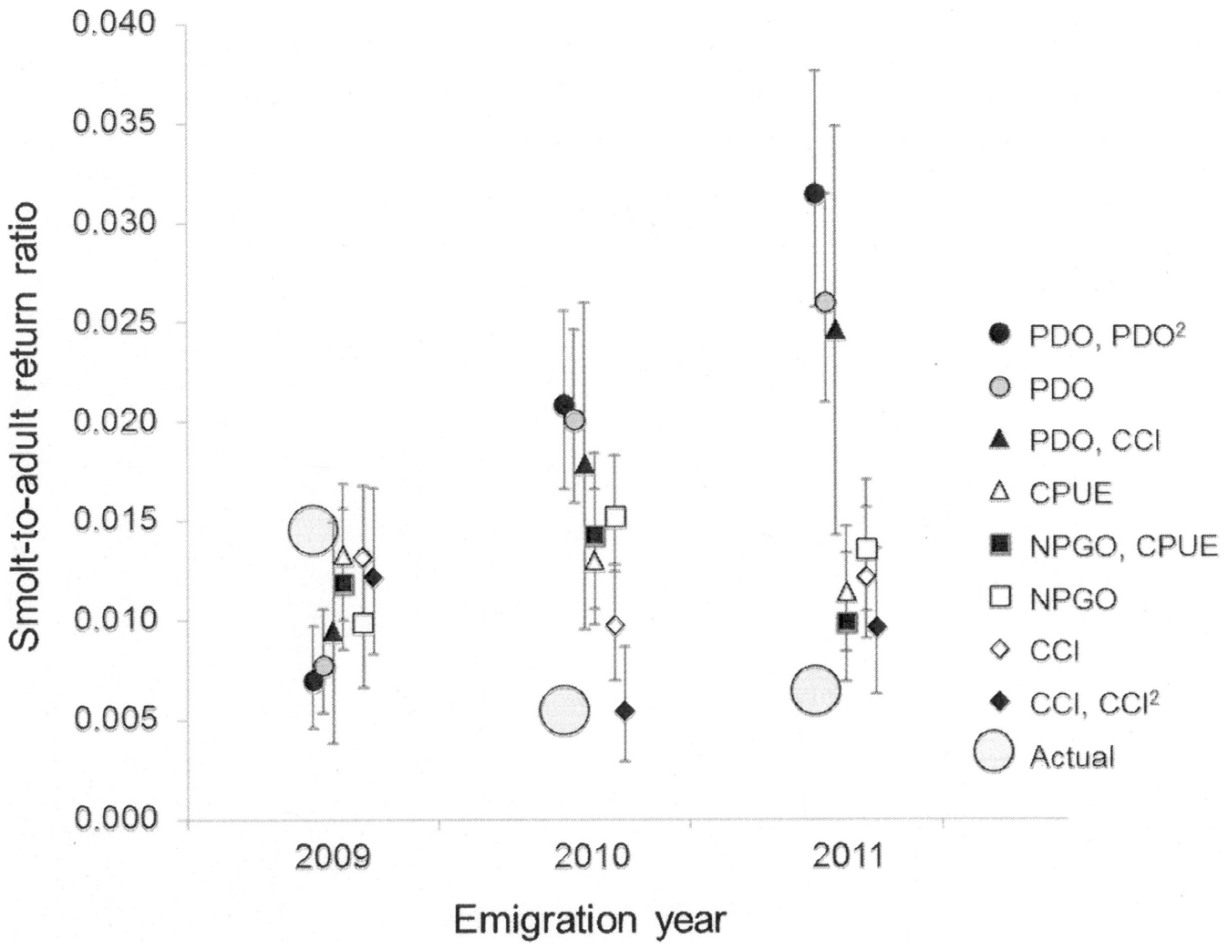
Model comparison for Snake River sp/su Chinook salmon survival. Observed and predicted smolt-to-adult return ratios (SAR) for Snake River sp/su Chinook salmon in emigration years 2009–2010 based on the top eight models presented in text. Mean prediction and the upper and lower 95% confidence intervals are presented for each model (see Table 4 for additional details). The “Actual” SAR values for 2009 and 2010 were obtained from http://www.fpc.org/. The 2011 SARs was estimated based on the relationship between SARs and adult returns of spring/summer Chinook salmon to Lower Granite Dam at a -2-yr lag (r = 0.815, 1998–2010).

Snake River sp/su Chinook salmon SARs have remained low in recent years. Therefore, we predicted SARs for the 2009–011 emigration years (Table S3 in File S1) using the family of models based on physical and biological variables described above to evaluate the utility of the variables identified in our hindcast models. There was a growing divergence among model predications across these recent years (Fig. 7). Models with only the PDO_7___9_ displayed greater error in prediction of SARs (50–448% of observed) compared with the NPGO (68–212% of observed) or local biological indices (CPUE  = 86–166% and CCI  = 80–241% of observed). The NPGO_4___6_, CPUE_6_, and CCI_6_ more successfully captured the recent variability in SARs (Fig. 7). Therefore, it appears that although the PDO index was remarkably informative in past years, other physical (NPGO) and biological indices (CCI_6_) and attributes of the juveniles (CPUE) provide a more accurate indication of survival variation in recent years.

## Discussion

We observed strong evidence for the growth-mortality hypothesis during early marine residence of Snake River sp/su Chinook salmon, which is a finding similar to the adjacent Mid-Upper Columbia River spring Chinook salmon genetic stock group [20]. In years during which adequate numbers of juveniles were collected for analysis, Snake River sp/su Chinook salmon SARs were positively correlated with early marine growth and size at capture. However, interannual variation in size at marine (brackish/ocean) entry was not significantly related to subsequent survival which indicates that, for juveniles that survived their first month at sea, early growth was a more important determinant of their subsequent survival than their size at initial marine entry. Our data indicate that there are survival advantages associated with faster marine growth and larger body size attained during the first 3–4 weeks at sea, which are potentially related to reduced over-winter mortality [16]. It is important to note that our approach did not examine selective mortality during in-river migration. We did not evaluate whether juveniles that are larger when they initiate downstream migration survive better, although there is evidence that this is true [24], [25]. Size-selective mortality in-river would result in more uniform sizes at marine entry followed by high variation in growth that could influence survival, potentially during the subsequent winter. Our data reveal such a pattern with relatively uniform sizes at marine entry (mean  = 134 mm FL with coefficient of variation (CV)  = 14%) and greater variation in early marine growth (CV = 46%). Size-selective mortality requires individual variation in size and there may be multiple periods in the life history when size-selective mortality can occur, such as during in-river migration and during the first ocean winter.

There was relatively weak support for the match–mismatch hypothesis, which is also similar to our finding for the Mid-Upper Columbia River spring Chinook salmon genetic stock group [20]. However, 80% of our juveniles were of hatchery origin, and their timing of marine entry can be influenced by hatchery management practices, such as release timing and transport (barging) protocols, in addition to natural variation in migration behavior. In general, juveniles consistently emigrated after the physical spring transition but there was minimal evidence that survival was greater if marine entry occurred longer after either the biological or physical transition. The lack of a significant relationship between survival and marine entry across years does not mean that, across evolutionary time scales, emigration timing has no influence on survival. Rather, we interpret this result to indicate that, under current management practices, there is only weak evidence for a relationship between survival and annual mean time of marine entry for juveniles that survived their first month at sea. Furthermore, it is important to note that there could be intra-annual variation in survival related to emigration timing [25], and survival advantages associated with timing may have been more evident under more protracted juvenile emigration, such as occurred historically [57].

There were some intriguing differences between the analyses that included a reduced number of years due to inadequate numbers of ocean-caught juveniles (1999–2000, 2002–2004, 2006–2008) and those analyses that encompassed all years (1998 to 2008). For years in which juvenile attributes were examined, their marine growth, size at capture, and subsequent survival were well-described by a suite of variables. In these years (1999–2000, 2002–2004, 2006–2008), variables indicative of conditions within the ocean basin (PDO and NPGO), local environment (plume area and copepod community composition), and juvenile abundance (CPUE) were all significantly correlated with survival (Table 2). However, when the years 1998, 2001, and 2005 were included in the analysis, the PDO was the most informative metric, and yielded a model that was >6 times more likely than other models, such as those with the NPGO or CPUE. These differences among models indicate that when juveniles can be examined after their initial 3–4 weeks in the ocean, certain attributes, such as size and growth, provide a strong indication of year-class strength. It is important to note that, with the exception of 2005, the SARs associated with those low juvenile collection years were not the lowest in our time series (Table S3 in File S1). However, those low catch years were rather unique: 1998 and 2005 were considered the worst ocean conditions for salmon growth and survival (http://www.nwfsc.noaa.gov/research/divisions/fe/estuarine/oeip/g-forecast.cfm) and 2001 had the lowest Columbia River flow and plume size. These observations indicate that the reduced statistical importance of the plume metrics when all years could be included in the analysis may be because certain plume conditions (i.e., large volume/area) may be important but not wholly adequate for good juvenile salmon survival. Therefore, for years with moderate ocean conditions, plume conditions are related to survival. However, when there are very poor ocean conditions, such as 1998 and 2005, the relative importance of the plume in relation to survival is minimized.

The ability to understand and predict fluctuations in marine fish populations has been a primary research focus for >100 years [3], [8]. The ultimate goal of identifying a suite of parameters that is relatively easy to measure and provides robust forecasts of abundance has proven elusive. Initial success in identifying likely mechanistic linkages, such as prey abundance and distribution [4], [21], [22], environmental thresholds or windows associated with high survival [5], or promising combinations of environmental correlates [49], [58], [59], often fail to result in viable predictive models for extended periods of time. However, the prospect of gaining a mechanistic understanding that forms the basis for more robust model development continues to motivate researchers. In this analysis, we did not seek to optimize our ability to predict recent survival (2009–2011 emigration years); rather we compared how well our top hindcast models performed in forecasting. The top hindcast model, based only on the PDO, substantially overestimated survival, potentially by >400% given the expected SARs for the 2011 cohort, but the two models with variables indicative of lower trophic levels (CCI) and juvenile abundance (CPUE) yielded the most accurate predictions, yet still overestimated recent survival by >170%. The use of model-averaged predictions has received increased attention recently and may prove useful [53], [60]. However, given the high weight of the PDO (−0.692) in our family of models, a model-averaged estimate would still have substantially over-estimated SARs for emigration years 2010 and likely for 2011. The fact that the best predictor of recent survival was the catch of juveniles in June (CPUE) demonstrates that the acquisition of biological information on a population after significant mortality events, or critical periods, may provide some of the most accurate indicators of changes in regulatory mechanisms. However, effectively integrating such information into management structures remains an important challenge.

Burke et al. [61] examined the relationships between adult returns of sp/su Chinook salmon to Ice Harbor Dam on the Snake River and a suite of physical and biological variables. They also found evidence for bottom-up, growth-mediated influences on survival and highlighted the importance of basin-scale indices, particularly the PDO, but they cautioned that such basin-scale relationships can be regime-dependent. The mechanisms regulating productivity and abundance can vary across climate regimes and, thus, predictive population models may be “regime-specific” [62], [63], [64]. Interestingly, the recent low survival of the 2010 and 2011 Snake River sp/su Chinook salmon cohorts (2012 and 2013 adult returns) occurred during a period of strongly negative PDO values. Therefore, even within regimes, the relationship between survival and basin-scale indicators can vary substantially and should be interpreted with caution.

The interannual patterns in size at marine entry, early marine growth, migration rate, and size at capture that we observed for the Snake River sp/su Chinook salmon yearlings are very similar to those observed for the Mid-Upper Columbia River spring Chinook salmon yearlings [20]. For example, annual mean size at marine entry ranged from 127–150 mm FL for the Snake River and from 126–156 mm FL for the Mid-Upper Columbia River stock. Mean size at capture was also similar (147–169 mm FL vs. 147–179 mm FL, for the Snake River and Mid-Upper Columbia River stock groups, respectively) [20]. Given that the only consistent difference was that the Snake River yearlings entered the ocean an average of 7–10 days later than Mid-Upper Columbia River yearlings, the small differences in mean size could be related to duration of marine residence. Other studies focused on these two populations also reported similarities: Rechisky et al. [13] compared early marine survival of yearling Snake River and yearling spring Chinook salmon from the Mid-Columbia River using acoustic tags in 2006, 2008, and 2009 and reported a high level of covariation in early marine survival between these two interior Columbia populations. They suggest that the cause of the consistently lower overall survival for Snake River sp/su Chinook salmon when compared to Mid-Columbia spring Chinook salmon may occur north of southern Vancouver Island, BC, which was the northern extent of their detection array. The lower survival of the Snake River sp/su Chinook salmon compared with the Mid-Columbia River spring Chinook salmon may be, at least in part, due to conditions further north and later in the life history. However, it is notable the two models that most accurately predicted recent SARs for Snake River sp/su Chinook salmon included indices of conditions within Northern California Current coastal waters, cohort abundance (CPUE) and the copepod community (CCI), which indicates that local conditions are important for understanding overall survival. Furthermore, the observation that juvenile CPUE in both May and June were positively correlated with SARs across years from 1999 to 2008 (0.759 and 0.855, respectively) provides additional evidence that conditions at marine entry or during very early marine residence influence subsequent survival [8], [9].

Organismal-level studies focused on changes in size, growth, or condition of individuals before and after critical periods in the life history can provide valuable insight into likely mechanisms of mortality [65], [66], [67]. Our approach, which combined field, genetic, and otolith-derived information, provided novel information on early marine residence in an ESA-protected population. We determined that juvenile abundance and size during early marine residence and local (plume area) and basin-scale (PDO) indicators were all good indicators of subsequent survival (r>0.85). In the absence of information on juvenile attributes, basin-scale indicators accounted for a lower but still substantial amount of the variation in survival (r>0.70). Although the low survival of Snake River sp/su Chinook salmon population may be related to factors within the river system and/or events that occur later in the life history, indices of cohort abundance and the copepod community within coastal waters remained the most informative of the available indicators in recent years. Future efforts to gain a mechanistic understanding of the population productivity of anadromous fishes will continue to benefit from organismal-level explorations across the life history.

## Supporting Information

File S1 **Tables S1–S3. Table S1. Juvenile spring/summer Snake River Chinook salmon with PIT tags included in the study.** Year of emigration and date and size at tagging are reported. The otolith-derived estimates for size at marine entry (FL_ME_) and duration of time at liberty prior to marine entry (Release to ME) are included with estimated mean in-river migration rate (In-river), date of marine entry (ME) and the date and location of final detection along the Columbia River hydropower system. **Table S2. Comparison between marked and unmarked Snake River yearling sp/su Chinook salmon.** Mean (SE) size at marine entry, marine growth rate, date of marine entry, marine migration rate (body length per second), and size at capture by emigration year. **Table S3. Annual values for model parameters.** Smolt-to-adult return ratios (SAR) for Snake River spring/summer Chinook salmon; NPGO_4___6_ =  mean value from April to June; PDO_7___9_ =  mean value from July to September; CPUE_6_ =  catch of yearling Chinook (fish km^−1^) in June; and CCI_6_ =  Copepod Community Index in June are included.
(DOCX)

## References

[1] Botsford LW (2001) Physical influences on recruitment to California Current invertebrate populations on multiple scales. ICES J Mar Sci 58: 1081–1091.

[2] Fogarty MJ, Sissenwine MP, Cohen EB (1991) Recruitment variability and the dynamics of exploited marine populations. Trends Ecol Evol 6: 241–246. 21232469 10.1016/0169-5347(91)90069-A

[3] Lehodey P, Alheit J, Barange M, Baumgartner T, Beaugrand G, et al (2006) Climate variability, fish, and fisheries. J Clim 19: 5009–5030.

[4] Lasker R (1974) Field criteria for survival of anchovy larvae: the relation between inshore chlorophyll maximum layers and successful first feeding. Fish Bull US 73: 453–462.

[5] Cury P, Roy C (1989) Optimal environmental window and pelagic fish recruitment success in upwelling areas. Can J Fish Aquat Sci 46: 670–680.

[6] Peterman RM, Bradford MJ (1987) Wind speed and mortality rate of a marine fish, the northern anchovy (*Engraulis mordax*). Science 235: 354–356. 17750387 10.1126/science.235.4786.354

[7] Peterman RM, Bradford MJ, Lo NCH, Methot RD (1988) Contribution of early life stages to interannual variability in recruitment of northern anchovy (*Engraulis mordax*). Can J Fish Aquat Sci 45: 8–16.

[8] Hjort J (1914) Fluctuations in the great fisheries of northern Europe. Rapports et Proces-verbaux des Reunions, Conseil international pour l'Exploration de la Mer 20: 1–228.

[9] Houde ED (2008) Emerging from Hjort's Shadow. J Northw Atl Fish Sci 41.

[10] Hickey B, Geier S, Kachel N, MacFadyen AF (2005) A bi-directional river plume: The Columbia in summer. Cont Shelf Res 25: 1631–1656.

[11] Ford MJ (editor) (2011) Status review update for Pacific salmon and steelhead listed under the Endangered Species Act: Pacific Northwest. U.S. Dept. Commerce. Available: http://noaa.ntis.gov/view.php?pid=NOAA:ocn775807530. Accessed 2013 Oct 1.

[12] Tuomikoski J, McCann J, Chockley B, Schaller H, Haeseker, et al. (2013) Comparative survival study (CSS) of PIT-tagged spring/summer Chinook and summer steelhead. Comparative Survival Study Oversight Committee and Fish Passage Center. Available: http://www.fpc.org/documents/CSS/CSS_2013_Annual_Report_rev1b.pdf. Accessed 2014 Mar 1.

[13] Rechisky EL, Welch DW, Porter AD, Jacobs-Scott MC, Winchell PM (2013) Influence of multiple dam passage on survival of juvenile Chinook salmon in the Columbia River estuary and coastal ocean. Proc Natl Acad Sci USA 110(17): 6883–6888. 23576733 10.1073/pnas.1219910110 PMC3637724

[14] Budy P, Thiede GP, Bouwes N, Petrosky CE, Schaller H (2002) Evidence linking delayed mortality of Snake River salmon to their earlier hydrosystem experience. N Amer J Fish Manage 22: 35–51.

[15] Haeseker S, McCann JA, Tuomikoski J, Chockley B (2012) Assessing freshwater and marine environmental influences on life-stage-specific survival rates of Snake River spring–summer Chinook salmon and steelhead. Trans Amer Fish Soc 141: 121–138.

[16] Beamish RJ, Mahnken C (2001) A critical size and period hypothesis to explain natural regulation of salmon abundance and the linkage to climate and climate change. Prog Oceanogr 49: 423–437.

[17] Pearcy WG (1992) Ocean Ecology of Pacific Salmonids. Seattle, WA: Washington Sea Grant. 179 p.

[18] Anderson JT (1988) A review of size dependent survival during pre-recruit stages of fishes in relation to recruitment. J NW Atlantic Fish Sci 8: 55–66.

[19] Sogard SM (1997) Size-selective mortality in the juvenile stage of teleost fishes: a review. Bull Mar Sci 60: 1129–1157.

[20] Tomaro L, Teel DJ, Peterson WP, Miller JA (2012) Early marine residence of Columbia River spring Chinook salmon: when is bigger better? Mar Ecol Prog Ser 452: 237–252.

[21] Cushing DH (1975) Marine Ecology and Fisheries. London, UK: Cambridge University Press. 292 p.

[22] Cushing DH (1990) Plankton production and year-class strength in fish populations: an update of the match/mismatch hypothesis. Adv Mar Biol 26: 249–293.

[23] Achord S, Sandford BP, Hockersmith EH, Nesbit MG, Dumdei ND, et al. (2011) Monitoring the migrations of wild Snake River spring/summer Chinook salmon juveniles, 2009–2010. Report of the National Marine Fisheries Service to the Bonneville Power Administration. Portland, OR. pp. 123. Available: http://www.nwfsc.noaa.gov/assets/26/7387_08012012_122716_Achord.et.al.2012-rev.pdf.Accessed 2013 Oct 1.

[24] Zabel RW, Achord A (2004) Relating size of juveniles to survival within and among populations of Chinook salmon. Ecology 85: 795–806.

[25] Scheuerell MD, Zabel RW, Sandford BP (2009) Relating juvenile migration timing and survival to adulthood in two species of threatened Pacific salmon (*Oncorhynchus* spp.). J Appl Ecol 46: 983–990.

[26] Myers JM, Kope RG, Bryant GJ, Teel D, Lierheimer LJ, et al. (1998) Status Review of Chinook Salmon from Washington, Idaho, Oregon, and California. NOAA Technical Memorandum NMFS-NWFSC-35. Available: http://www.nwr.noaa.gov/publications/status_reviews/salmon_steelhead/chinook/sr1998-chinook1.pdf. Accessed 2013 Oct 1.

[27] Teel DJ, Baker C, Kuligowski DR, Friesen TA, Shields B (2009) Genetic stock composition of subyearling Chinook salmon in seasonal floodplain wetlands of the lower Willamette River, Oregon. Trans Amer Fish Soc 138: 211–217.

[28] Barnett-Johnson R, Teel DJ, Casillas E (2010) Genetic and otolith isotopic markers identify salmon populations in the Columbia River at broad and fine geographic scales. Environ Biol Fish 89: 533–546.

[29] Seeb LW, Antonovich A, Banks MA, Beacham TD, Bellinger MR, et al (2007) Development of a standardized DNA database for Chinook salmon. Fisheries 32: 540–552.

[30] Kalinowski ST, Manlove KR, Taper ML (2007) ONCOR A computer program for genetic stock identification. Department of Ecology, Montana State University, Bozeman, MT Available: www.montana.edu/kalinowski/Software/ONCOR.htm. Accessed 2013 Oct 1.

[31] Rannala B, Mountain JL (1997) Detecting immigration by using multilocus genotypes. Proc Natl Acad Sci 94: 9197–9201. 9256459 10.1073/pnas.94.17.9197 PMC23111

[32] Miller JA (2009) The effects of temperature and water concentration on the otolith incorporation of barium and manganese in black rockfish *Sebastes melanops* . J Fish Biol 75: 39–60. 20738481 10.1111/j.1095-8649.2009.02262.x

[33] Kent A, Ungerer C (2006) Analysis of light lithophile elements (Li, Be, B) by laser ablation ICP-MS: Comparison between magnetic sector and quadrupole ICP-MS. Amer Mineral 91: 1401–1411.

[34] Miller JA (2009) The effects of temperature and water concentration on the otolith incorporation of barium and manganese in black rockfish *Sebastes melanops* . J Fish Biol 75: 39–60. 20738481 10.1111/j.1095-8649.2009.02262.x

[35] Neilson JD, Geen GH (1982) Otoliths of Chinook salmon (*Oncorhynchus tshawytscha*) - daily growth increments and factors influencing their production. Can J Fish Aquat Sci 39: 1340–1347.

[36] Miller JA, Gray A, Merz J (2010) Quantifying the contribution of juvenile migratory phenotypes in a population of Chinook salmon *Oncorhynchus tshawytscha* . Mar Ecol Prog Ser 408: 227–240.

[37] Miller JA, Butler VL, Simenstad CA, Backus DH, Kent AJR (2011) Life history variation in upper Columbia River Chinook salmon (*Oncorhynchus tshawytscha*): a comparison using modern and ∼500-year-old archaeological otoliths. Can J Fish Aquat Sci 68: 603–617.

[38] Burla M, Baptista AM, Zhang YL, Frolov S (2010) Seasonal and interannual variability of the Columbia River plume: A perspective enabled by multiyear simulation databases. J Geophys Res-Oceans 115 (C2).

[39] Zhang YL, Baptista AM (2008) SELFE: A semi-implicit Eulerian-Lagrangian finite-element model for cross-scale ocean circulation. Ocean Model 21: 71–96.

[40] Zhang YL, Baptista AM, Myers EP (2004) A cross-scale model for 3D baroclinic circulation in estuary-plume-shelf systems: I. Formulation and skill assessment. Cont Shelf Res 24: 2187–2214.

[41] Mantua NJ, Hare SR (2002) The Pacific Decadal Oscillation. J Ocean 58: 35–44.

[42] Hare SR, Mantua NJ, Francis RC (1999) Inverse production regimes: Alaska and West Coast Pacific salmon. Fisheries 24(1): 6–14.

[43] Hare SR, Mantua NJ (2000) Empirical evidence for North Pacific regime shifts in 1977 and 1989. Prog Oceanogr 47: 103–145.

[44] Di Lorenzo E, Schneider N, Cobb KM, Franks PJS, Chhak K, et al (2008) North Pacific Gyre Oscillation links ocean climate and ecosystem change. Geophys Res Lett 35: 6.

[45] Hooff RC, Peterson WT (2006) Copepod biodiversity as an indicator of changes in ocean and climate conditions of the northern California current ecosystem. Limnol Oceanogr 51: 2607–2620.

[46] Morgan CA, Peterson WT, Emmett RL (2003) Onshore-offshore variations in copepod community structure off the Oregon coast during the summer upwelling season. Mar Ecol Prog Ser 249: 223–236.

[47] Keister JE, Di Lorenzo E, Morgan CA, Combes V, Peterson WT (2011) Zooplankton species composition is linked to ocean transport in the Northern California Current. Glob Change Biol 17: 2498–2511.

[48] Checkley DM, Barth JA (2009) Patterns and processes in the California Current System. Prog Oceanogr 83: 49–64.

[49] Logerwell EA, Mantua N, Lawson PW, Francis RC, Agostini VN (2003) Tracking environmental processes in the coastal zone for understanding and predicting Oregon coho (*Oncorhynchus kisutch*) marine survival. Fish Oceanogr 12: 554–568.

[50] Peterson WT, Schwing FB (2003) A new climate regime in northeast Pacific ecosystems. Geo Res Lett 30(17).

[51] Healey MC (1991) Life history of Chinook salmon (*Oncorhynchus tshawytscha*). In: Groot C, Margolis L, editors. Pacific salmon life histories: UBC Press. pp. 313–393.

[52] Pyper BJ, Peterman RM (1998) Comparison of methods to account for autocorrelation in correlation analysis of fish data. Can J Fish Aquat Sci 55: 2127–2140.

[53] Anderson DR (2008) Model based inference in the life sciences: a primer on evidence. New York, NY: Springer-Verlag. 184 p.

[54] Prentice EF, Flagg TA, McCutcheon CS (1990) Feasibility of using implantable passive integrated transponder (PIT) tags in salmonids. In: Parker NC, Giorgi AE, Heidinger RC, Jester Jr DB, Prince ED et al., editors. Fish-marking techniques.Bethesda, Maryland: American Fisheries Society, Symposium 7. pp. 317–322.

[55] Elsdon TS, Gillanders BM (2003) Relationship between water and otolith elemental concentrations in juvenile black bream *Acanthopagrus butcheri* . Mar Ecol Prog Ser 260: 263–272.

[56] Miller JA (2011) Effects of water temperature and barium concentration on otolith composition along a salinity gradient: implications for migratory history. J Exp Mar Biol Ecol 405: 42–52.

[57] Achord S, Mathews GM, Johnson OW, Marsh DM (1996) Use of passive integrated transponder (PIT) tags to monitor migration timing of Snake River Chinook salmon smolts. N Amer J Fish Manage 16: 302–313.

[58] Checkley DMJ, Raman S, Maillet GL, Mason KM (1988) Winter storm effects on the spawning and larval drift of a pelagic fish. Science 335: 356–348.

[59] Lawson PW, Logerwell EA, Mantua NJ, Francis RC, Agostini VN (2004) Environmental factors influencing freshwater survival and smolt production in Pacific Northwest coho salmon (*Oncorhynchus kisutch*). Can J Fish Aquat Sci 61: 360–373.

[60] Rupp D, Wainwright T, Lawson P, Peterson W (2012) Marine environment-based forecasting of coho salmon (*Oncorhynchus kisutch*) adult recruitment. Fish Oceanogr 21: 1–19.

[61] Burke BJ, Peterson WT, Beckman BR, Morgan C, Daly EA, et al. (2013) Multivariate models of adult Pacific salmon returns. PLOS ONE 8(1). 10.1371/journal.pone.0054134 PMC3543311 23326586

[62] Anderson PJ, Piatt JF (1999) Community reorganization in the Gulf of Alaska following ocean climate regime shift. Mar Ecol Prog Ser 189: 117–123.

[63] Bailey KM (2000) Shifting control of recruitment of walleye pollock *Theragra chalcogramma* after a major climatic and ecosystem change. Mar Ecol Prog Ser 198: 215–224.

[64] Shanks A, Roegner GC, Miller J (2010) Using megalopae abundance to predict future commercial catches of Dungeness crabs (*Cancer magister*) in Oregon. Cal Coop Ocean Fish Invest Rep 51: 106–118.

[65] Meekan MG, Vigliola L, Hansen A, Doherty P, Halford A, et al (2006) Bigger is better: size-selective mortality throughout the life history of a fast-growing clupeid, *Spratelloides gracilis* . Mar Ecol Prog Ser 317: 237–244.

[66] Miller JA, Teel DJ, Baptista A, Morgan CA (2013) Disentangling bottom-up and top-down effects on survival during early ocean residence in a population of Chinook salmon (*Oncorhynchus tshawytscha*). Mar Ecol Prog Ser 70: 617–629.

[67] Woodson L, Wells BK, Johnson RC, Weber P, MacFarlane RB, et al (2013) Evaluating selective mortality of juvenile Chinook salmon (*Oncorhynchus tshawytscha*) across years of varying ocean productivity. Mar Ecol Prog Ser 487: 163–175.

