# Supplementary material for: Assessing the Relative Importance of Local and Regional Processes on the Survival of a Threatened Salmon Population
Source: PLoS One. 2014 Jun 12;9(6):e99814. doi: 10.1371/journal.pone.0099814 (PMC4055704; doi:10.1371/journal.pone.0099814)
Supplement: File S1 — Tables S1–S3. Table S1. Juvenile spring/summer Snake River Chinook salmon with PIT tags included in the study. Year of emigration and date and size at tagging are reported. The otolith-derived estimates for size at marine entry (FLME) and duration of time at liberty prior to marine entry (Release to ME) are included with estimated mean in-river migration rate (In-river), date of marine entry (ME) and the date and location of final detection along the Columbia River hydropower system. Table S2. Comparison between marked and unmarked Snake River yearling sp/su Chinook salmon. Mean (SE) size at marine entry, marine growth rate, date of marine entry, marine migration rate (body length per second), and size at capture by emigration year. Table S3. Annual values for model parameters. Smolt-to-adult return ratios (SAR) for Snake River spring/summer Chinook salmon; NPGO4_6 = mean value from April to June; PDO7_9 = mean value from July to September; CPUE6 = catch of yearling Chinook (fish km−1) in June; and CCI6 = Copepod Community Index in June are included. (DOCX) [file pone.0099814.s001.docx]

| **Fish** | **Migration Year** | **Tag (DOY)** | **Tag FL (mm)** | **FL_ME_ (mm)** | **Release to ME (d)** | **In-river (km/d)** | **ME (DOY)** | **Final detection**  **DOY (locale)** |
| --- | --- | --- | --- | --- | --- | --- | --- | --- |
| 1 | 2003 | 51 | 121 | 132 | 71 | 7 | 147 | 144 (JDJ) |
| 2 | 2008 | 35 | na | 147 | 69 | 8 | 145 | 97 (RPJ) |
| 3 | 2007 | 36 | na | nd | 68 | 8 | 142 | 142 (BCC) |
| 4 | 2008 | 36 | na | 152 | 66 | 8 | 142 | 133 (GOJ) |
| 5 | 2008 | 37 | 122 | 142 | 65 | 8 | 142 | 130 (MCJ) |
| 6 | 2003 | 43 | 119 | nd | 62 | 8 | 141 | 144 (JDJ) |
| 7 | 2008 | 39 | na | 138 | 62 | 8 | 138 | 142 (ICH) |
| 8 | 2008 | 36 | na | 142 | 66 | 8 | 140 | 141 (LMJ) |
| 9 | 2008 | 129 | 148 | 158 | 21 | 25 | 151 | 130 (LGR) |
| 10 | 2007 | 299 | 94 | 145 | 28 | 28 | 131 | 125 (JDJ) |
| *11* | *2004* | *149* | *103* | *109* | *10* | *52* | *159* | *155 (JDJ)* |
| *12* | *2006* | *128* | *97* | *118* | *10* | *52* | *138* | *128 (LOL)* |
| *13* | *2008* | *131* | *138* | *129* | *9* | *58* | *141* | *132 (LGR)* |
| *14* | *2003* | *150* | *115* | *108* | *3* | *174* | *154* | *152 (GOJ)* |

**Table S1. Juvenile spring/summer Snake River Chinook salmon with PIT tags included in the study.** Year of emigration and date and size at tagging are reported. The otolith-derived estimates for size at marine entry (FL_ME_) and duration of time at liberty prior to marine entry (Release to ME) are included with estimated mean in-river migration rate (In-river), date of marine entry (ME) and the date and location of final detection along the Columbia River hydropower system. “DOY” indicates day of year. “na’ indicates that no data were available. Interrogation information for Fish 11-14 (*italicized*) indicates likely in-river transport via barge. Abbreviations are for juvenile fish rearing, collection or detection facilities at: JDJ = John Day Dam; RPJ = Rapid River Hatchery; BCC = Bonneville Dam; GOJ = Little Goose Dam; MCJ = McNary Dam; ICH = Ice Harbor Dam; LMJ = Lower Monumental Dam; LOL = Lolo Creek, Idaho screw trap; LGR = Lower Granite Dam.

**Table S2. Comparison between marked and unmarked Snake River yearling sp/su Chinook salmon**. Mean (SE) size at marine entry, marine growth rate, date of marine entry, marine migration rate (body length per second), and size at capture by emigration year. Smaller samples size indicate the number of juveniles that were included in otolith analyses and displayed adequate otolith growth in marine environment to estimate size at marine entry and marine growth rate. Size at capture was based on all ocean collections. *Bold text indicates significant difference between groups (p < 0.05). ND indicates sample size was too small to calculate a mean value.

| **Mark** | **Size at marine entry (FL, mm)** | **Marine growth**  **(% d^-1^, mm)** | **Day of marine entry** | | **Marine migration rate (bl s^-1^)** | **n** | **Size at capture**  **(FL, mm)** | **n** |
| --- | --- | --- | --- | --- | --- | --- | --- | --- |
| 1999 | | | | | | | | |
| Y | 134.0 (6.3) | 0.78 (0.12) | 127 (4.6) | | 0.45 (0.10) | 7 | 158.5 (2.1) | 122 |
| N | 128.3 (4.3) | 0.68 (0.06) | 120 (3.0) | | 0.40 (0.04) | 20 | 160.9 (4.1) | 48 |
| 2000 | | | | | | | | |
| Y | 136.4 (5.5) | 0.92 (0.12) | 112 (3.1) | | 0.33 (0.05) | 7 | 166.57 (5.1) | 28 |
| N | 130.2 (6.4) | 0.66 (0.21) | 119 (4.5) | | 0.41 (0.02) | 3 | 159.93 (7.7) | 14 |
| 2002 | | | | | | | | |
| Y | 144.7 (2.4) | 0.51 (0.14) | 126 (2.6) | | 0.24 (0.03) | 7 | 154.25 (5.1) | 28 |
| N | 159.7 (14.7) | 0.40 (0.27) | 131 (7.9) | | 0.47(0.14) | 4 | 156.71 (9.0) | 7 |
| 2003 | | | | | | | | |
| Y | 136.7 (5.6) | 0.61 (0.06) | 131 (4.0) | | 0.49 (0.07) | 14 | 152.00 (3.8) | 40 |
| N | 115.4 (8.4) | 0.77 (0.09) | 142 (5.7) | | 0.84 (0.10) | 4 | 131.00 (4.5) | 15 |
| 2004 | | | | | | | | |
| Y | 138.8 (8.8) | 0.58 (0.12) | 131 (7.0) | | 0.54 (0.14) | 8 | 145.71 (7.5) | 24 |
| N | ND | ND | ND | | ND | 1 | 130.00 (2.0) | 3 |
| 2006 | | | | | | | | |
| Y | 126.9 (5.4) | 0.61 (0.10) | 112 (3.1) | | 0.56 (0.13) | 10 | 147.82 (2.0) | 90 |
| N | 125.9 (9.1 | 0.41 (0.17) | 119 (4.5) | | 1.20 (0.10) | 2 | 136.36 (11.1) | 11 |
| 2007 | | | | | | | | |
| Y | 143.8 (2.9) | 0.54 (0.04) | 127 (3.4) | | 0.51 (0.06) | 17 | 149.41 (1.9) | 88 |
| N | 127.8 (5.8) | 0.68 (0.14) | 128 (4.6) | | 0.54 (0.09) | 6 | 139.57 (5.2) | 14 |
| 2008 | | | | | | | | |
| Y | 140.2 (2.8) | 0.69 (0.06) | | 133 (3.2) | ***0.51 (0.05)** | 22 | ***165.9 (1.6)** | 175 |
| N | 111.6 (2.2) | 0.99 (0.07) | | 140 (4.0) | **0.85 (0.09)** | 14 | **141.9 (3.8)** | 24 |

**Table S3. Annual values for model parameters.** Smolt-to-adult return ratios (SAR) for Snake River spring/summer Chinook salmon; NPGO_4_6_ = mean value from April to June; PDO_7_9_ = mean value from July to September; CPUE_6_ = catch of yearling Chinook (fish km^-1^) in June; and CCI_6_ = Copepod Community Index in June are included.

| **Emigration year** | **SAR** | **NPGO_4_6_** | **PDO_7_9_** | **CPUE_6_** | **CCI_6_** |
| --- | --- | --- | --- | --- | --- |
| 1998 | 0.012 | 0.306 | -0.490 | 0.264 | 1.092 |
| 1999 | 0.024 | 1.719 | -1.050 | 1.271 | -1.061 |
| 2000 | 0.017 | 2.229 | -1.030 | 1.040 | -1.156 |
| 2001 | 0.013 | 2.169 | -1.150 | 0.435 | -0.811 |
| 2002 | 0.009 | 1.202 | 0.240 | 0.845 | -1.075 |
| 2003 | 0.003 | 0.933 | 0.617 | 0.630 | -0.257 |
| 2004 | 0.005 | 0.370 | 0.680 | 0.424 | -0.231 |
| 2005 | 0.002 | -1.149 | 0.150 | 0.128 | 1.142 |
| 2006 | 0.007 | -0.056 | -0.413 | 0.690 | -0.051 |
| 2007 | 0.010 | 0.910 | 0.307 | 0.864 | -0.855 |
| 2008 | 0.027 | 1.546 | -1.640 | 2.559 | -1.146 |
| 2009 | 0.015 | 0.791 | 0.027 | 0.970 | -0.838 |
| 2010 | 0.005 | 1.689 | -1.310 | 0.890 | -0.240 |
| 2011 | ^1^0.006 | 1.438 | -1.797 | 0.460 | -0.676 |

^1^The 2011 SARs was estimated based on the relationship between SARs and adult returns of sp/su Chinook salmon to Lower Granite Dam (LGD) at a -2-yr lag (r = 0.816, 1998-2010) and the 2013 sp/su adult return to LGD (43,454 adults).
